# 5-HT4-Receptors Modulate Induction of Long-Term Depression but Not Potentiation at Hippocampal Output Synapses in Acute Rat Brain Slices

**DOI:** 10.1371/journal.pone.0088085

**Published:** 2014-02-05

**Authors:** Matthias Wawra, Pawel Fidzinski, Uwe Heinemann, Istvan Mody, Joachim Behr

**Affiliations:** 1 Department of Psychiatry and Psychotherapy, Charité Universitätsmedizin Berlin, Berlin, Berlin, Germany; 2 Institute of Neurophysiology, Charité Universitätsmedizin Berlin, Berlin, Berlin, Germany; 3 Exzellenzcluster NeuroCure, Charité Universitätsmedizin Berlin, Berlin, Berlin, Germany; 4 Department of Neurology, Charité Universitätsmedizin Berlin, Berlin, Berlin, Germany; 5 Department of Neurology, David Geffen School of Medicine, University of California Los Angeles, Los Angeles, California, United States of America; 6 Department of Psychiatry, Psychotherapy and Psychosomatics, Ruppiner Kliniken, Neuruppin, Brandenburg, Germany; Centre national de la recherche scientifique, University of Bordeaux, France

## Abstract

The subiculum is the principal target of CA1 pyramidal cells and mediates hippocampal output to various cortical and subcortical regions of the brain. The majority of subicular pyramidal cells are burst-spiking neurons. Previous studies indicated that high frequency stimulation in subicular burst-spiking cells causes presynaptic NMDA-receptor dependent long-term potentiation (LTP) whereas low frequency stimulation induces postsynaptic NMDA-receptor-dependent long-term depression (LTD). In the present study, we investigate the effect of 5-hydroxytryptamine type 4 (5-HT4) receptor activation and blockade on both forms of synaptic plasticity in burst-spiking cells. We demonstrate that neither activation nor block of 5-HT4 receptors modulate the induction or expression of LTP. In contrast, activation of 5-HT4 receptors facilitates expression of LTD, and block of the 5-HT4 receptor prevents induction of short-term depression and LTD. As 5-HT4 receptors are positively coupled to adenylate cyclase 1 (AC1), 5-HT4 receptors might modulate PKA activity through AC1. Since LTD is blocked in the presence of 5-HT4 receptor antagonists, our data are consistent with 5-HT4 receptor activation by ambient serotonin or intrinsically active 5-HT4 receptors. Our findings provide new insight into aminergic modulation of hippocampal output.

## Introduction

Activity-dependent changes in synaptic strength are thought to be one of the cellular mechanisms underlying learning and memory [1]–[3]. Two different forms of long-lasting synaptic plasticity have been characterized, long-term potentiation (LTP) and long-term depression (LTD) [4]. Both forms of synaptic plasticity have been intensively studied in the CA1 and CA3 areas of the hippocampus, based on their established role in formation of spatial memory [4].

The subiculum (Sub) is the principal target of CA1 pyramidal cells and the major hippocampal output structure [5], as subicular pyramidal cells project to numerous cortical and subcortical structures [5], [6]. Pyramidal cells in the subiculum have been characterized according to their firing properties as regular-spiking (RS) and burst-spiking (BS) cells. In response to depolarizing current injection, BS cells fire a burst of action potentials (AP) followed by single APs whereas RS neurons fire a train of single action potentials [7], [8]. In most studies, BS cells outnumber RS cells in rodents by approximately two to one [6], [8] (but see [9]). *In vivo* and *in vitro* studies failed to induce LTD in field potential recordings [10], [11]. Intracellular recordings, however, showed that low frequency stimulation (LFS) induces LTD in BS cells but LTP in RS cells [12]. This finding indicates that in field potential recordings, LTD in BS cells seems to be masked by a simultaneous LTP in RS cells.

The subiculum receives a strong serotonergic input from the raphe nuclei [13]–[15]. *In vivo* experiments have shown that different serotonergic receptor subtypes have a distinct impact on learning and memory performance under various experimental conditions (for reviews, see [16]–[18]). The 5-hydroxytryptamine type 4 (5-HT4) receptor is ubiquitously expressed in the hippocampus and positively coupled to intracellular adenylate cyclase 1 (AC1) [19]–[23]. Although it has been shown that activation of 5-HT4 receptors modulates network plasticity in the CA1 and the dentate gyrus of the hippocampus *in vitro* [24] and *in vivo* [25], [26], little is known about the effect of this receptor on synaptic plasticity at hippocampal output synapses. In the present study we demonstrate that 5-HT4 receptor activation enhances LTD whereas blockade of this receptor prevents induction of LTD in subicular BS cells.

## Materials and Methods

All procedures were performed in accordance with national and international guidelines (EC Directive 86/609/EEC for animal experiments) and were approved by the local health authority (Landesamt für Gesundheit und Soziales Berlin). Male Wistar rats (4-6 weeks) were decapitated under deep ether anesthesia and the brains were quickly removed. Horizontal slices (400 µm) containing the hippocampal formation and the entorhinal cortex (EC) were prepared using a VT1200S vibroslicer (Leica Microsystems GmbH, Germany). The tissue was prepared in ice-cold, oxygenated (95% O2, 5% CO2) artificial cerebrospinal fluid (ACSF) composed of (in mM): NaCl 129, Na_2_PO_4_ 1.25, NaHCO_3_ 26, KCl 3, CaCl_2_ 1.6, MgSO_4_ 1.8, glucose 10 at a pH of 7.4, and stored for later use in an interface chamber at 34°C. As in all experiments GABA_A_ receptor-mediated transmission was blocked by bicuculline (5-10 µM), the concentration of MgSO_4_ and CaCl_2_ was elevated to 4 mM each in the recording medium in order to prevent epileptiform discharges [27]–[29]. In previous work we showed that increased Mg^2+^- and Ca^2+^-levels as well as blockade of inhibition are not related to bursting [30], [31].

Single cell recordings in the pyramidal cell layer (middle-to-distal portion) of the subiculum were performed at 32°–34°C with sharp microelectrodes (50-80 MΩ) filled with 2.5 M potassium acetate.

Recordings were performed in current-clamp bridge mode using a SEC10LX amplifier (NPI Electronic, Tamm, Germany), an ITC-16 interface (Instrutech Corp., Great Neck, NY, USA) and TIDA software (Version 5.050, HEKA GmbH, Lambrecht, Germany). Signals were low-pass filtered at 3 kHz, sampled and processed at 10 kHz.

For characterization of cellular discharge and membrane properties, hyper- and depolarizing current steps (200 ms, -0.1 to 1.2 nA) were applied. Excitatory postsynaptic potentials (EPSPs) were evoked by constant voltage stimulation (100 µs stimulus, 1 to 10 V) of CA1 efferents with an ACSF-filled patch pipette in stratum oriens of CA1. To avoid activation of the trisynaptic hippocampal loop, CA1-Sub-EC minislices were used. The amplitudes of evoked EPSPs were set to 30–50% of the maximum response for LTP experiments and to 50–80% for LTD experiments. T_rise_ and T_decay_ were defined as the time between 20 and 80% of the rising phase and the time between 100 and 37% of the decaying phase of the EPSP, respectively. Analyses for T_rise_ and T_decay_ were performed with R software (version 2.15.2) and the minpack.lm library (version 1.1–6) [32], [33].

For activation of the 5-HT4 receptor we used the potent and highly selective partial agonist RS 67333 (5–10 µM) [34]–[36]. For block of the 5-HT4 receptor we used the potent and selective antagonists RS 39604 [36], [37] and GR 113808 [36], [38].

Recordings of isolated NMDA receptor-mediated EPSPs were performed in the presence of the AMPA receptor antagonist CNQX (30 µM) and the GABA_B_ receptor antagonist CGP55845 (20 µM). For inhibiting the cAMP dependent protein kinase (PKA), slices were pre-incubated with H 89 (10 µM) for a minimum of 60 minutes.

Synaptic responses were evoked every 10 s. For induction of synaptic plasticity, three different stimulation protocols were used: low-frequency stimulation (LFS) consisting of 900 paired pulses (50 ms inter-stimulus interval) applied at 1 Hz, subthreshold high-frequency stimulation (stHFS) consisting of one train of 10 pulses applied at 40 Hz, and high-frequency stimulation (HFS) consisting of four trains of 100 pulses at 100 Hz with an inter-train interval of 9 seconds. Changes in synaptic strength were measured for at least 30 min after termination of the stimulation protocol and were expressed either as a percentage of the normalized baseline amplitudes at 20–25 min after the stimulation protocol or as the difference in the initial EPSP slope which was defined as the amplitude between 20% and 80% of the EPSP divided by the time. Unless otherwise stated, Student's t-test (paired and non-paired) or analysis of variance (ANOVA) with post-hoc TukeyHSD-test [32] were used where appropriate. Statistical significance level was set to p<0.05 and is marked in figures by asterisks (* p<0.05, ** p<0.01, *** p<0.001).

Analysis of the paired-pulse ratio (PPR) was applied to obtain evidence for presynaptic or postsynaptic modifications of synaptic transmission [39]. The PPR was defined as the response ratio (second EPSP amplitude/first EPSP amplitude) to a pair of stimuli given at an interstimulus interval of 50 ms.

Except for CNQX (Ascent Scientific, UK) all substances were obtained from Tokris (UK) and dissolved and stored as stock solutions at 1000 times the end concentration in distilled water, with the exception of RS 39604 and CGP 55845 which were dissolved in DMSO and GR 113808 which was dissolved in 1 eq. HCl. Except for RS 67333 and RS 39604 (see Results), all drugs were applied throughout the entire course of the experiment and for at least 5 min prior to recording.

## Results

### Effects of 5-HT4 receptors on synaptic and intrinsic properties of subicular pyramidal neurons

**Figure 1:**
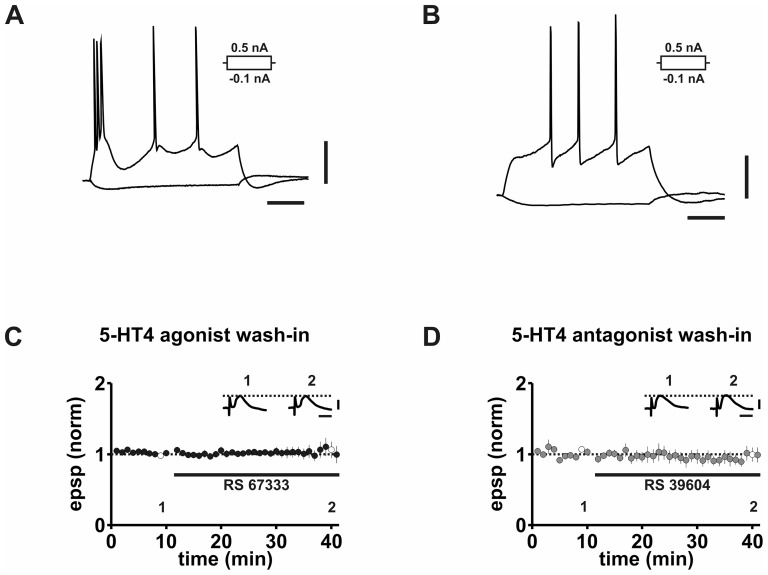
Effect of 5-HT4 receptors on synaptic and intrinsic properties of subicular BS neurons. A: Voltage responses of a burst-spiking subicular neuron upon depolarizing and hyperpolarizing current pulses. B: Voltage responses of a regular-spiking subicular neuron to depolarizing and hyperpolarizing current pulse. C: The 5-HT4 receptor agonist RS 67333 does not modulate EPSP responses during 30 minutes of wash-in. D: The 5-HT4 receptor antagonist RS 39604 does not alter EPSP responses during 30 minutes of wash-in. Scale bars: 20 mV and 50 ms (B); 2 mV and 20 ms (C1, D1).

Subicular pyramidal cells are divided in two main groups, burst spiking (BS) and regular spiking (RS) non-bursting cells [7], [8], [40]–[42]. BS neurons are predominant in the subiculum [6] and upon depolarizing current injections generate a burst of action potentials followed by single action potentials (Fig. 1A), whereas RS neurons generate a series of single action potentials (Fig. 1B). We obtained and analyzed sharp microelectrode recordings from 105 burst-spiking neurons. The mean resting membrane potential was -65.0 ± 0.4 mV and the mean input resistance 31.1 ± 0.7 MΩ. Neither the 5-HT4 receptor agonist RS 67333 nor the 5-HT blocker RS 39604 altered intrinsic properties (Table 1). There was also no detectable effect on CA1 stimulus-induced excitatory postsynaptic potentials in CA1-Sub minislices (Fig. 1C/D).

**Table 1:** Synaptic and membrane properties of BS-cells in the subiculum before and after application of 5-HT4 receptor ligands.

	RS 67333 (10 µM)		RS 39604 (25 µM)	
**EPSP (% of Baseline)**	102.0±8.3 (n = 7, p = 0.85)		93.1±8.0 (n = 6, p = 0.48)	
	**Baseline**	**Wash-in**	**Baseline**	**Wash-in**
**Rise-time (ms)**	3.4±0.4	3.3±0.4	3.8±0.6	3.5±0.5
	(n = 6, p = 0.54)		(n = 4, p = 0.26)	
**Decay-time (ms)**	10.9±1.3	11.0±1.4	14.5±1.6	14.1±1.4
	(n = 6, p = 0.79)		(n = 5, p = 0.42)	
**RMP (mV)**	−73.0±3.1	−73.4±3.7	−64.6±1.6	−65.6±2.0
	(n = 5, p = 0.83)		(n = 6, p = 0.12)	
**R_in_ (M**Ω**)**	29.7±6.4	30.6±7.6	40.6±2.5	39.2±3.5
	(n = 5, p = 0.67)		(n = 6, p = 0.44)	

Data given as means ± SEM.

Regular spiking neurons had a mean resting potential of −69.3 ± 1.0 mV and a mean input resistance of 34.0 ± 2.2 MΩ (n = 12). Like in BS cells, activation or blockade of the 5-HT4 receptors had no effect on synaptic or intrinsic properties (Table S1, Fig. S1A/B). In the present study, we subsequently focused on the effect of 5-HT4 receptor-activation on synaptic plasticity in BS cells.

### Effect of 5-HT4 receptors on LTP

**Figure 2:**
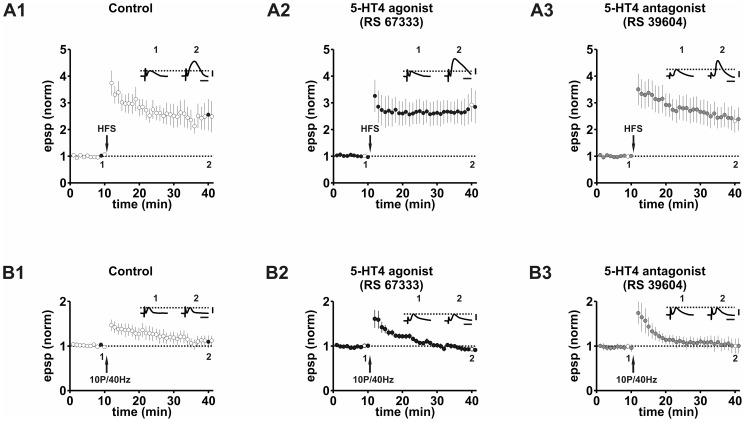
Effect of 5-HT4 receptors on LTP. A1: HFS induces LTP under control conditions. A2: The 5-HT4 receptor agonist RS 67333 does not alter LTP. A3: The 5-HT4 receptor antagonists RS 39604 does not modulate LTP. B1: stHFS induces PTP but not LTP under control conditions. B2: The 5-HT4 receptor agonist RS 67333 failed to prime LTP after stHFS. B3: The 5-HT4 receptor antagonist RS 39604 failed to facilitate the induction of LTP after stHFS. Scale bars: 2 mV and 20 ms.

In control experiments, HFS of CA1 fibers in stratum oriens in area CA1 induced a cellular LTP of 245.4 ± 41.4% of baseline response (n = 7, p<0.01, Fig. 2A1). Application of the 5-HT4 receptor agonist RS 67333 (266.4±42.9% of baseline response, n = 8, p<0.01, Fig. 2A2), or of the receptor antagonist RS 39604 (251.2±44.0% of baseline response, n = 8, p<0.01, Fig. 2A3) had no significant effects either on the induction or on the expression of LTP in BS cells (ANOVA, one-way, F(2,20) = 0.055, p = 0.95; see also Table S2). In the presence of the 5-HT4 receptor agonist RS 67333, we observed a more stabilized LTP during its initial phase in comparison to control experiments. The amount of LTP 30 min after HFS, however, was not significantly different.

To determine whether activation of 5-HT4 receptors has a facilitating effect on synaptic potentiation, we applied a subthreshold conditioning high-frequency stimulation protocol (stHFS) that failed to induce LTP in control experiments (112.7±12.7% of baseline response, n = 7, p = 0.39, Fig. 2B1) [43]. LTP could still not be induced by stHFS in the presence of the 5-HT4 agonist RS 67333 (103.8±5.1% of baseline response, n = 8, p = 0.55, Fig. 2B2), or in the presence of the receptor antagonist RS 39604 (107.6±11.4% of baseline, n = 6, p = 0.49, Fig. 2B3). Comparison of all three groups showed no statistically significant differences (ANOVA, one-way, F(2,18) = 0.187, p = 0.83).

### 5-HT4 receptors modulate LTD

**Figure 3:**
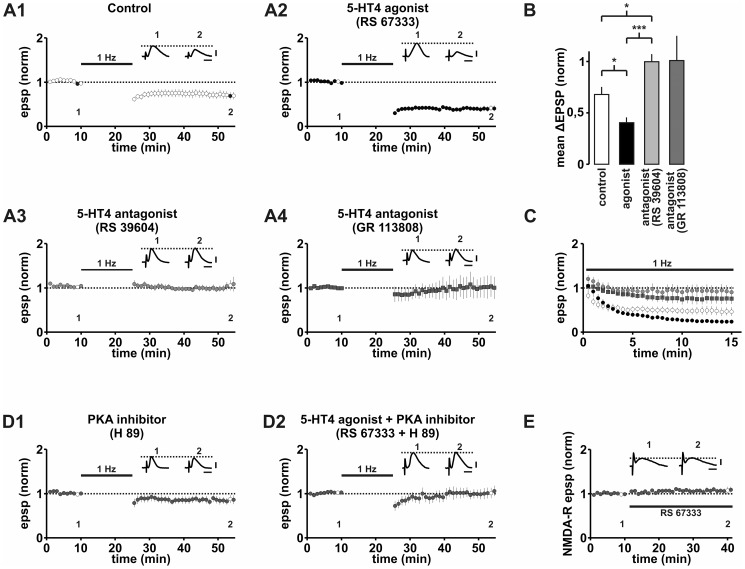
5-HT4 receptors modulate LTD. A1: LFS induces LTD under control conditions. A2: Activation of 5-HT4-receptors significantly facilitates LTD. A3/4: Blockade of 5-HT4-receptors through RS 39604 or GR 113808 prevented LTD. B: Summary of changes in synaptic strength illustrated in A1-4. The antagonist GR 13808 is not included in the ANOVA. C: Averaged time courses of normalized EPSP responses during LFS. Control: white circles, RS 67333: black circles, RS 39604: gray circles, GR 113808: dark gray squares. D1: The PKA inhibitor H 89 prevented LTD under control conditions. D2: H 89 prevented LTD even in the presence of the 5-HT4 receptor agonist RS 67333. E: A 5-HT4 receptor agonist failed to modulate NMDA receptor mediated EPSPs during 30 minutes wash-in. Scale bars: 2 mV and 20 ms (A,D), 1 mV and 20 ms (E).

As in our previous study [12], LFS caused LTD of synaptic potentials to 67.8±7.4% of the baseline response (n = 7, p<0.01, Fig. 3A1). In presence of the 5-HT4 receptor agonist RS 67333, LTD was significantly enhanced to 40.5±4.8% of the baseline response (n = 7, p<0.001, Fig. 3A2). In contrast, LTD was blocked in the presence of two different 5-HT4 receptor antagonists (RS 39604: 99.8±7.2% of the baseline response, n = 8, p = 0.95, Fig. 3A3; GR 113808: 100.6±24.3% of the baseline response, n = 6, p = 0.98, Fig. 3A4). Differences in LTD between control, and in the presence of the 5-HT4 agonist RS 67333 and the 5-HT4 antagonist RS 39604 were significant (ANOVA, one-way, F(2,19) = 17.572, p<0.001; post-hoc: control vs. RS 67333: p<0.05, control vs. RS 39604: p<0.05, RS 67333 vs. RS 39604: p<0.001, Fig. 3B). Comparable results were obtained when analyzing the initial slope of EPSPs instead of EPSP amplitudes (Table S2).

The induction of LTD in subicular BS neurons depends on NMDA receptor activation and the increase of postsynaptic Ca^2+^ concentration [12]. To determine whether 5-HT4 receptor activation alters NMDA receptor mediated potentials, we investigated the effect of 5-HT4 receptor activation on isolated NMDA receptor-mediated EPSPs at resting membrane potential. The 5-HT4 receptor agonist RS 67333 did not alter the amplitudes (108.4±7.9% of baseline, n = 5, p = 0.32, Fig. 3E), and rise or decay times (T_rise_ = 11.0±1.1 ms vs. 12.0±1.1 ms after wash-in, n = 5, p = 0.12; T_decay_ = 28.4±3.1 ms vs. 30.4±2.9 ms after wash-in, n = 6, p = 0.22) of isolated NMDA receptor EPSPs. To study the effect of 5-HT4 receptors on the induction phase of LTD, we analyzed EPSP amplitudes during LFS. Comparison of the three groups (control, RS 67333, RS 39604) showed significant differences (ANOVA, one-way, F(2,19) = 9.811, p<0.01). Under control condition and in experiments with the 5-HT4 agonist RS 67333, we observed a significant decline of EPSP amplitudes during the course of LFS which was not statistically different between the two groups (control: 46.5±9.4% of baseline response, n = 7, p<0.01; RS 677333: 23.6±3.2% of baseline response, n = 7, p<0.001; control vs. RS 67333 p = 0.35; Fig. 3C). In contrast, there was no significant depression if the 5-HT4 receptor was blocked by the antagonist RS 39604 (90.7±13.8% of baseline response, n = 8, p = 0.55; RS 39604 vs. RS 67333: p<0.01; RS 39604 vs. control: p<0.03; Fig. 3C). When the 5-HT4 receptor was blocked by GR 113808 we recorded a small but not significant depression of EPSP amplitudes during LFS (75.2±12.1% of baseline response, n = 5, p = 0.14, Fig. 3C, not included in the ANOVA). These data support the notion, that 5-HT4 receptor-activation affects synaptic plasticity at the short term scale. Consistent with our previous study [12], the analysis of the paired-pulse ratio before and after induction of LTD showed no difference and provided no evidence for a presynaptic expression of LTD (control: PPR 1.25±0.27, n = 7, p = 0.26; RS67333: PPR 1.17±0.8, n = 7, p = 0.55; RS39604: PPR 1.11±0.07, n = 8, p = 0.17).

5-HT4 receptors are positively coupled to AC1 and therefore, their activation elevates intracellular cAMP levels. We studied the effect of inhibition of cAMP dependent PKA on LTD induction and found that LTD was strongly reduced in the presence of the PKA inhibitor H 89 (89.2±5.2% of baseline, n = 6, p = 0.12, Fig. 3D1). In addition, the facilitated LTD in the presence of the 5-HT4 receptor agonist RS 67333 was likewise prevented when the PKA inhibitor H 89 was applied (97.1±9.5% of baseline, n = 7, p = 0.80, Fig. 3D2).

## Discussion

In the present study, we show that 5-HT4 receptors modulate activity-dependent LTD but not LTP in subicular BS cells. We demonstrate that activation of 5-HT4 receptors by the agonist RS 67333 increases LTD, while blockade of the receptor by the antagonists RS 39604 or GR 113808 prevents LTD. Our data indicate that 5-HT4 receptor activation facilitates postsynaptic LTD. The paired-pulse ratios did not change after LTD induction, providing no evidence for a presynaptic expression of LTD. Analyses of EPSP amplitudes during the course of LFS demonstrate that 5-HT4 receptors have also an effect on short term depression (STD). Since STD is a known presynaptic effect due to depletion of presynaptic vesicles in the course of the stimulation [39], our results indicate that 5-HT4 receptors have independent effects on the presynaptic (STD) and postsynaptic (LTD) function. Application of 5-HT4 agonists or antagonists has no effect on BS cells' resting membrane potential, input resistance, EPSP amplitude or EPSP kinetics.

In our experiments, we did not observe a clear-cut effect of 5-HT4 modulating agents on suprathreshold or subthreshold LTP induction. Though we observed a slight difference in the initial time course of EPSP amplitudes between the 5-HT4 agonist and the two other experimental conditions (control, 5-HT4 antagonist), the amount of LTP was not significantly different 30 minutes after induction.

We have to consider, that RS 67333 like other 5-HT4 agonist may interact with other receptors as well. Hence, the observed effect might not be mediated solely by an action on 5-HT4 receptors. Since LTD was blocked in the presence of 5-HT4 receptor antagonists, however, our data suggest that 5-HT4 receptors are active, possibly due to the presence of ambient serotonin [24] or to its activity-dependent release. Alternatively, brain specific splice variants of the 5-HT4 receptor with high intrinsic activity might be affected by application of 5-HT4 antagonists with inverse agonist activity like GR 113808 [44], [45]. Since inverse agonist activity is not shown for RS 39604 [45], 5-HT4 receptor activation by ambient serotonin seems to be more likely.

Our data indicate that 5-HT4 receptor activation modulates LTD which is blocked by the PKA-inhibitor H 89. 5-HT4 receptors are positively coupled to AC1 through a G protein (G_s_). Though H 89 is known to block various kinases, and other signaling cascades cannot entirely be excluded, it is feasible that the modulation of LTD by 5-HT4 receptors is mediated by the AC1-cAMP-PKA-cascade [19]–[23], [46]. Previous reports showed that LTD in subicular BS cells depends on NMDA receptors and requires the increase of postsynaptic Ca^2+^ [12]. Although PKA can potentiate NMDA receptor mediated currents by phosphorylation [47], our results provide no evidence that 5-HT4 receptor activation modulates the kinetic of NMDA receptor-mediated EPSPs in BS cells.

In various experimental tasks including the Morris water maze, the social olfactory recognition task, the olfactory associative discrimination task or the two-trail recognition task, *in vivo* application of 5-HT4 agonists improves the performance of the animals [34], [48]–[52] supporting an important role of 5-HT4 receptors in learning and memory. There is also growing evidence that 5-HT4 receptors may play a role in Alzheimer's disease and might be a promising target for treatment of memory impairments [53]–[56]. Interestingly, Kemp and Manahan-Vaughan demonstrated that blockade or activation of 5-HT4 receptors modulates LTD in the CA1 *in vivo* [26] suggesting that the modulatory effect is not restricted to the subiculum. As in the present study, activation of 5-HT4 receptors did not modulate LTP [26], but in sharp contrast to our findings, activation of the receptor blocked LTD and blockade of the receptor lowered the threshold for LTD induction. Notably, the same group showed that exposure to a novel object-place configuration lowered the threshold for the induction of LTD in CA1 [57]. This facilitation could be blocked by *in vivo* administration of a 5-HT4 receptor agonist before exposure to the novel object-place configuration [57].

A major difference between CA1 and subicular pyramidal neurons resides in their discharge behavior. Whereas most CA1 pyramidal neurons exhibit regular-spiking behavior [58], the majority of subicular pyramidal neurons fire high-frequency bursts of action potentials in response to current injection. As burst-spiking has been shown to be important for neuronal signaling and plasticity [59], [60], the abundance of burst-spiking neurons in the subiculum suggests that they may be critical to the encoding and processing of hippocampal output information. *In vivo* experiments indicate that the subiculum operates earlier than the hippocampus in a limited time frame of 10–15 s to encode and maintain new information in a highly accurate and specific manner [61]. This process is followed by an increasing participation of CA1 in the encoding and retrieval of this information. These data support the hypothesis that the subiculum occupies a pivotal position in the hippocampal memory system, where it receives raw information directly from peri- and postrhinal cortices and processes information via the entorhinal–hippocampal polysynaptic circuit [62]. The subiculum may thus act as a detector and distributor of sensory information that takes into account the novelty and relevance of signals arriving from CA1 [62], [63]. The contrasting effect of 5-HT4 receptor activation on LTD in the CA1 and subiculum supports the hypothesis of different but complementary information processing of these two hippocampal output regions [61].

## Supporting Information

Figure S1 **Effect of 5-HT4 receptors on synaptic and intrinsic properties of subicular RS neurons.** A1, A2: The 5-HT4 receptor agonist RS 67333 does not modulate EPSP responses, input resistance (R_in_) or resting membrane potential (RMP) during 30 minutes of wash-in. B1, B2: The 5-HT4 receptor antagonist RS 39604 does not alter EPSP responses, input resistance (R_in_) or resting membrane potential (RMP) during 30 minutes of wash-in. Scale bars: 2 mV and 20 ms.
(PDF)

Table S1 **Synaptic and membrane properties of RS-cells in the subiculum before and after application of 5-HT4 receptor ligands.**
(PDF)

Table S2 **Normalized mean delta of the initial slope of EPSPs of BS-cells in the subiculum after HFS or LFS in control condition and after application of 5-HT4 receptor ligands.**
(PDF)
