# Supplementary figures and images for: 5-HT4-Receptors Modulate Induction of Long-Term Depression but Not Potentiation at Hippocampal Output Synapses in Acute Rat Brain Slices
Source: PLoS One. 2014 Feb 5;9(2):e88085. doi: 10.1371/journal.pone.0088085 (PMC3914937; doi:10.1371/journal.pone.0088085)

**A1****5-HT4 agonist wash-in**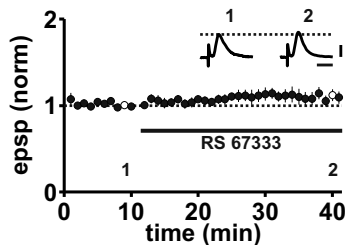**A2**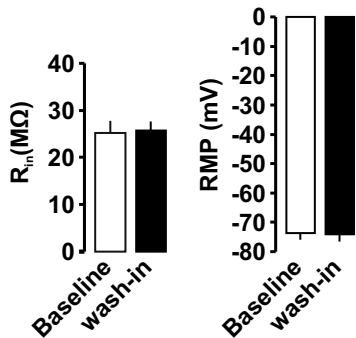**B1****5-HT4 antagonist wash-in**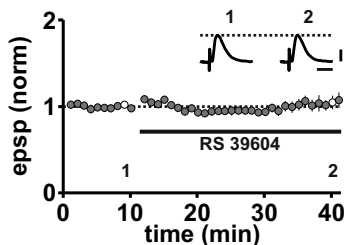**B2**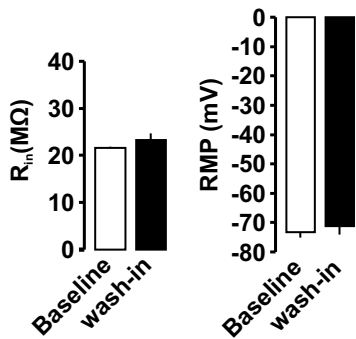

Supplement: Figure S1 — Effect of 5-HT4 receptors on synaptic and intrinsic properties of subicular RS neurons. A1, A2: The 5-HT4 receptor agonist RS 67333 does not modulate EPSP responses, input resistance (Rin) or resting membrane potential (RMP) during 30 minutes of wash-in. B1, B2: The 5-HT4 receptor antagonist RS 39604 does not alter EPSP responses, input resistance (Rin) or resting membrane potential (RMP) during 30 minutes of wash-in. Scale bars: 2 mV and 20 ms. (PDF) [file pone.0088085.s001.pdf]
