# Supplementary material for: 5-HT4-Receptors Modulate Induction of Long-Term Depression but Not Potentiation at Hippocampal Output Synapses in Acute Rat Brain Slices
Source: PLoS One. 2014 Feb 5;9(2):e88085. doi: 10.1371/journal.pone.0088085 (PMC3914937; doi:10.1371/journal.pone.0088085)
Supplement: Table S1 — Synaptic and membrane properties of RS-cells in the subiculum before and after application of 5-HT4 receptor ligands. (PDF) [file pone.0088085.s002.pdf]

Table S1. Synaptic and membrane properties of RS-cells in the subiculum before and after application of 5-HT4 receptor ligands.

	**RS 67333 (10 µM)**		**RS 39604 (25 µM)**	
**EPSP (% of Baseline)**	109.7±7.8 (n=6, p=0.31)		101.1±5.6 (n=6, p=0.85)	
	**Baseline**	**Wash-in**	**Baseline**	**Wash-in**
**Rise-time (ms)**	3.4±0.2	3.6±0.2	2.9±0.2	2.7±0.1
	(n=5, p=0.50)		(n=5, p=0.41)	
**Decay-time (ms)**	11.3±0.9	11.3±0.9	10.4±0.5	10.6±0.3
	(n=5, p=0.97)		(n=4, p=0.78)	
**RMP (mV)**	-73.5±2.2	-73.8±2.5	-73.0±1.8	-70.9±2.9
	(n=6, p=0.77)		(n=4, p=0.26)	
**R_{in} (MΩ)**	25.2±2.6	25.8±1.8	21.6±0.3	23.2±1.5
	(n=4, p=0.89)		(n=4, p=0.46)	

Data given as means ± SEM.
