# Supplementary material for: 5-HT4-Receptors Modulate Induction of Long-Term Depression but Not Potentiation at Hippocampal Output Synapses in Acute Rat Brain Slices
Source: PLoS One. 2014 Feb 5;9(2):e88085. doi: 10.1371/journal.pone.0088085 (PMC3914937; doi:10.1371/journal.pone.0088085)
Supplement: Table S2 — Normalized mean delta of the initial slope of EPSPs of BS-cells in the subiculum after HFS or LFS in control condition and after application of 5-HT4 receptor ligands. (PDF) [file pone.0088085.s003.pdf]

Table S2. Normalized mean delta of the initial slope of EPSPs of BS-cells in the subiculum after HFS or LFS in control condition and after application of 5-HT4 receptor ligands.

	**HFS**	**LFS**
**Control**	2.81±0.57 (n=7, p<0.01)	0.59±0.06 (n=6, p<0.01)
**Agonist (RS 67333)**	3.02±1.23 (n=7, p<0.05)	0.38±0.06 (n=6, p<0.01)
**Antagonist (RS 39604)**	2.73±0.39 (n=6, p<0.01)	0.94±0.04 (n=5, p=0.28)
**ANOVA (one-way)**	F(2,17)=0.026, p=0.98	F(2,14)=19.307, p<0.001
**Posthoc**	-	Control-RS 67333: p=0.08<br>Control-RS 39604: p<0.01<br>RS 67333-RS 39604: p<0.001

Data given as means ± SEM.
